# Structural and chemical characterization of MoO_2_/MoS_2_ triple-hybrid materials using electron microscopy in up to three dimensions†

**DOI:** 10.1039/d0na00806k

**Published:** 2020-12-29

**Authors:** Anna Frank, Thomas Gänsler, Stefan Hieke, Simon Fleischmann, Samantha Husmann, Volker Presser, Christina Scheu

**Affiliations:** Max-Planck-Institut für Eisenforschung GmbH, Independent Research Group Nanoanalytics and Interfaces Düsseldorf Germany a.frank@mpie.de c.scheu@mpie.de; INM – Leibniz Institute for New Materials Saarbrücken Germany; Department of Materials Science and Engineering, Saarland University Saarbrücken Germany; Materials Analytics, RWTH Aachen University Aachen Germany

## Abstract

This work presents the synthesis of MoO_2_/MoS_2_ core/shell nanoparticles within a carbon nanotube network and their detailed electron microscopy investigation in up to three dimensions. The triple-hybrid core/shell material was prepared by atomic layer deposition of molybdenum oxide onto carbon nanotube networks, followed by annealing in a sulfur-containing gas atmosphere. High-resolution transmission electron microscopy together with electron diffraction, supported by chemical analysis *via* energy dispersive X-ray and electron energy loss spectroscopy, gave proof of a MoO_2_ core covered by few layers of a MoS_2_ shell within an entangled network of carbon nanotubes. To gain further insights into this complex material, the analysis was completed with 3D electron tomography. By using *Z*-contrast imaging, distinct reconstruction of core and shell material was possible, enabling the analysis of the 3D structure of the material. These investigations showed imperfections in the nanoparticles which can impact material performance, *i.e.* for faradaic charge storage or electrocatalysis.

## Abstract 2

In-depth characterization of MoO_2_/MoS_2_ core/shell nanoparticles within a network of carbon nanotubes gives insights into the structure of the material and also possible degradation pathways.

## Graphical abstract

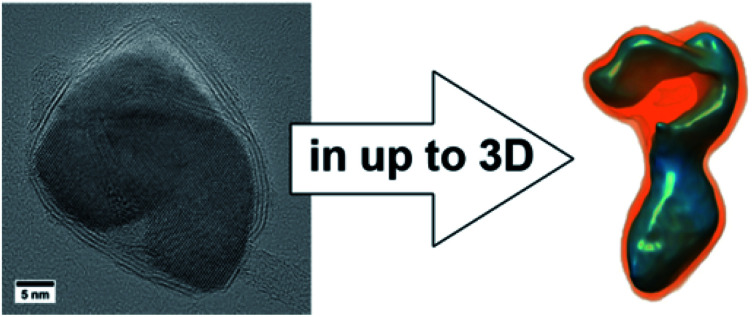

## 1 Introduction

Rising awareness for global warming and the increasing scarcity of fossil fuels has tremendously advanced global research efforts in renewable energy. Key enabling technology for the latter is electrochemical energy storage, which is also in high demand for advanced mobile communication and transportation applications. A promising material system for both intercalation- and conversion-type electrodes are hybrid materials, which combine carbon and metal oxides or sulfides on a nanoscale, leading to improved energy storage performance metrics.^1,2^ For example, the incorporation of metal oxide/sulfide nanoparticles into a conductive network can alleviate cycling stability issues by maintaining electrical percolation, while at the same time improving ion transport through the electrode and reduce diffusion limitations.^3,4^ Known hybrid electrode materials are highly conductive carbon nanomaterials, such as carbon nanotubes (CNT), carbon onions, or reduced graphene oxide decorated with transition metal oxides and/or sulfides that enable faradaic charge storage. Among the most studied faradaic materials in hybrid electrodes is molybdenum disulfide, where the hybridization with a carbon phase can significantly increase cycling stability and specific power.^5–11^ The use of core/shell morphologies can further improve the efficiency and stability of the material undergoing faradaic reactions.^12^ Hereby, the shell material can also be carbon, a metal, metal oxide, or sulfide.^12–14^

To prepare this kind of morphologies, the chosen synthesis must fulfill specific requirements. For example, deposition at lower temperatures and the ability to penetrate the whole surface area of the carbon support are essential. Different synthesis routes were successfully used already, like hydrothermal or direct conversion.^15,16^ Another well-suited synthesis approach is atomic layer deposition (ALD) with subsequent annealing treatment to create nanoparticles or thin film coatings.^1,7,17^ The crystallinity of the material can either be controlled by the deposition temperature or the annealing step, by which also the desired crystalline modification and phase can be influenced.^7^

A thorough characterization of these materials, down to the nanometer scale, is crucial to fully understand their behavior in electrochemical energy storage applications such as batteries, supercapacitors, hybrid capacitors, or electrocatalysis. A versatile method for analyzing nanostructures in great detail is (scanning) transmission electron microscopy ((S)TEM), which not only gives information about crystallinity and chemical composition but also provides insight into the 3D structure of the materials by methods like electron tomography.^18–21^ With 3D tomography, the core/shell architecture can be characterized, including, the thickness of the shell or the contact zone between the core and the shell. The tomography approach overcomes the limitation of the 2D projection of 3D objects in conventional imaging techniques and enables the correlation between structural properties of hybrid electrodes and their electrochemical energy storage performance.

As a model system where the 3D reconstruction is crucial, molybdenum oxide/molybdenum sulfide core/shell particles embedded within a network of CNTs were chosen. This triple-hybrid material is of particular interest for electrochemical applications, such as lithium-ion storage and electrocatalytic hydrogen evolution,^22–26^ but shows a high complexity that cannot be fully understood with conventional (S)TEM measurements and other analysis methods. For the material synthesis, first, molybdenum oxide was grown within the CNTs *via* ALD; then, a subsequent sulfidation step combined with the annealing treatment was used to prepare core/shell nanoparticles. A thorough electron microscopical characterization of up to three dimensions is presented.

## 2 Experimental

### 2.1 Synthesis of molybdenum oxide/molybdenum sulfide core/shell nanoparticles within a matrix of entangled carbon nanotubes

The ALD synthesis procedure of molybdenum oxide onto a network of CNTs followed by annealing in synthetic air and argon atmosphere was published elsewhere.^7^ In the present work, the procedure was modified by annealing of the material directly in a sulfurizing atmosphere to achieve the molybdenum oxide/molybdenum sulfide core/shell particles.

First, free-standing CNT film electrodes were prepared by dispersion of CNT powder (Nanocyl NC7000), vacuum filtration, and a drying step for 3 h at 120 °C. The final thickness of the CNT film electrodes was ≈50 μm. The as-obtained CNT film was placed into an OpAL system (open-load atomic layer deposition, Oxford Instruments) in a vertical alignment to enable deposition from both sides. The molybdenum oxide coating was applied by 100 ALD cycles which were sequences of molybdenum(vi)-carbonate (MoCO_6_, Pegasus Chemicals) as a metalorganic precursor (preheated to 60 °C and bubbled by the argon carrier gas into the chamber) and ozone as the reactant gas. One cycle consisted of a supply of MoCO_6_ for 20 s, 10 s purging with argon gas, 45 s of ozone, and a final 15 s pumping step.^7^ The temperature during deposition was maintained at 165 °C.

These samples were further sulfurized and heat-treated simultaneously. For this, they were placed into a crucible in a quartz tube positioned in the hot zone of a furnace heated to 550 °C. Located upstream of the electrodes, a crucible with elemental sulfur was placed, where a temperature of 200 °C was maintained. During annealing, the tube was flushed by a gas mixture of argon and hydrogen with flow rates of 50 sccm and 10 sccm (standard cubic centimeters per minute), respectively, to *in situ* generate hydrogen sulfide (H_2_S). The oven was heated at a rate of 30 °C min^−1^ to 550 °C, held for 15 min of sulfidation time, and subsequently cooled down to room temperature after the experiment. Hydrogen flow was maintained during the heating and holding periods; while cooling the tube was flushed solely by argon.

### 2.2 Characterization

X-ray diffraction (XRD) was carried out with a D8 Discover XRD from Bruker AXS (Cu Kα, 40 kV, 40 mA), with a Göbel mirror, a 0.5 mm point focus, and a VANTEC-500 detector. With the detector a range of about 20° 2*θ* was covered simultaneously per measurement step, leading to three measurement steps with the detector positioned at 20, 40, and 60° 2*θ* for 17 min each.

High-resolution TEM (HR TEM) was performed on a Thermo Scientific Titan Themis 300 microscope, equipped with a C_S_ image corrector, and operated at 300 kV. Calibration for electron diffraction was carried out using a Si standard and evaluated by comparison to literature data.^27^ For scanning TEM (STEM) a complementary Thermo Scientific Titan Themis microscope was used, which is equipped with a C_S_ probe corrector, a Gatan Quantum ERS energy filter, and a Bruker Super X-EDX detector. STEM imaging was conducted by using the attached high angle annular dark-field (HAADF) detector from Fischione. STEM imaging was done at an acceleration voltage of 300 kV while for both dual electron energy-loss spectroscopy (EELS) and energy-dispersive X-ray (EDX) measurements in STEM mode an acceleration voltage of 120 kV was used. EELS was performed with a dispersion of 0.1 eV and a spectral resolution of around 1 eV. For (S)TEM sample preparation, small pieces of the respective electrodes were ultrasonicated with a mixture of ethanol and deionized water (1 : 1) until a homogeneous suspension was formed. This suspension then was drop-cast onto carbon-coated gold grids and left to dry overnight. Different kinds of carbon coating were used, namely, continuous carbon coating or lacey carbon coating, to compare how the background influences the reconstruction of the electron tomography data. To avoid contamination effects while acquiring the tilt series, the samples were plasma cleaned for 30 s before inserting them into the microscope.

### 2.3 Electron tomography – acquisition and reconstruction

For the acquisition of the tilt series, a special single-tilt tomography holder from Thermo Scientific was used. Tilt angles ranged from −60° to +60°, with tilt increments of 5°. For the tilt series, the camera length for HAADF was lowered to avoid any residual diffraction contrast and achieve pure *Z*-contrast imaging conditions. Reconstruction of the 3D volume was done by aligning the image series *via* the TomoJ plugin^28^ for ImageJ and subsequent application of the simultaneous iterative reconstruction technique (SIRT)^29–31^ and well as refinement by the discrete algebraic reconstruction technique (DART)^18,32^ by an in-house MATLAB implementation.

## 3 Results and discussion

**Fig. 1:**
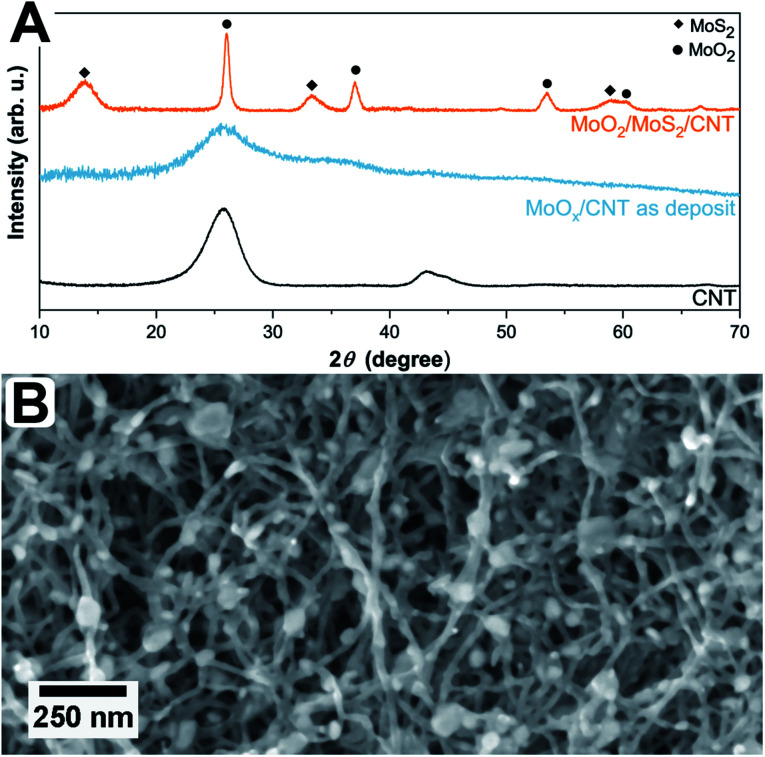
(A) XRD pattern of uncoated CNTs (bottom, black), as-deposited MoO_*x*_ on CNTs (middle, blue), and sulfurized MoO_2_/MoS_2_ on CNTs (top, orange). Peaks originating from MoS_2_ and MoO_2_ are marked with ◆ and ●, accordingly. (B) SEM top view image of MoO_2_/MoS_2_ core/shell nanoparticles within an entangled CNT network.

The simultaneous sulfurization and annealing treatment of the original amorphous MoO_*x*_ films deposited by ALD onto CNTs yields crystalline core/shell nanoparticles attached to the CNT support. In Fig. 1, the XRD pattern of the initial CNTs (black, bottom) is compared to the as-deposited MoO_*x*_/CNT (blue, middle) and the sulfurized MoO_2_/MoS_2_/CNT sample (orange, top). For the initial CNTs, only three broad and partly overlapping peaks at 26°, 42°, and 44° are visible, corresponding to the (002), (100), and (101) planes of graphite, respectively (AMCSD 0011247).^27^ After ALD deposition (sample as-deposited MoO_*x*_/CNT), the pattern appears very similar as only a thin layer of an amorphous coating of MoO_*x*_ was added. The pattern is showing a higher noise level compared to the pure CNTs as a lower amount of sample was measured. Finally, after annealing in H_2_S atmosphere, the pattern shows that the amorphous MoO_*x*_ material transformed into crystalline MoS_2_ and MoO_2_. The sharp peak with a high intensity occurring at ≈26° correspond to the (11−1) plane of MoO_2_, the other sharp peaks at ≈37°, 54°, and 60° have a lower intensity and originate from (111), (22−2) and (13−1) planes of the MoO_2_, respectively (MoO_2_ ICSD 80830).^33^ Other assigned peaks are at ≈14°, ≈33°, and ≈58°. Unfortunately, these reflections fit both hexagonal (ICSD 49801)^34^ and orthorhombic MoS_2_ (ICSD 38401)^35^ and do not allow for a conclusion on which modification is present. Generally, MoS_2_ has broader peaks in the XRD pattern compared to MoO_2_. The Scherrer equation^36^ relates a peak broadening reciprocally to the crystal size and, therefore, the observed broader signals of MoS_2_ hint to a smaller crystal size of MoS_2_ compared to MoO_2_ in the sample. This is in accordance to the TEM data discussed below which show that the number of atomic layers and perfection within the MoS_2_ shell is much lower than the crystal size of the MoO_2_ core. The peaks of the CNTs are not visible in this pattern, as the X-ray scattering amplitude of carbon is very weak. All shown pattern are normalized according to their intensity, such that the CNT peaks in the pattern of pure CNT and amorphous MoO_*x*_ on CNT are appearing intense. However, compared to the peaks of the crystalline MoO_2_/MoS_2_ the peak intensities of the carbon support material are very low and thus not visible.

**Fig. 2:**
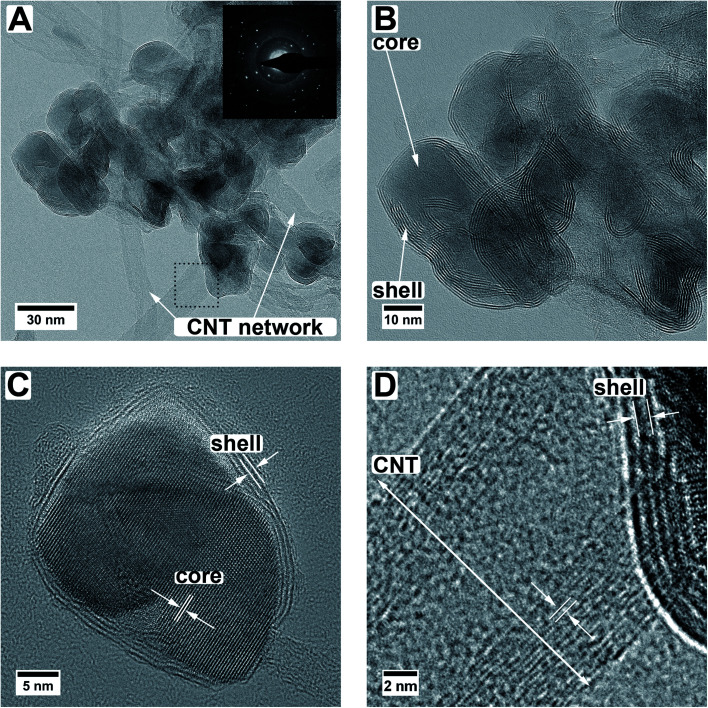
TEM images of the MoO_2_/MoS_2_ core/shell particles on multiwall carbon nanotubes. Panels A and B show the entangled core/shell/CNT network. The inset in (A) shows a diffraction pattern over some of the particles. Panel C displays an HR TEM image of a single particle. Panel D gives a higher magnification image of the area marked with a dotted square in panel A, demonstrating the different lattice distances of the multiwall CNTs *versus* the lattice distances of the shell material of the particles.

Fig. 1B shows a top view SEM overview image of the sample. It is visible, that the sample is composed of an entangled network of carbon nanotubes, within which small nanoparticles are visible. The particles are distributed inhomogeneously throughout the sample, resulting in areas with higher and areas with a lower number of particles. It can also be observed that the size of the particles varies but is around 30 nm. Investigated in more detail by TEM (Fig. 2A and B), the average size of the particles is 28 ± 10 nm. The large variation stems from the wide particle size range from values as low as 12 nm up to 62 nm. In some places, it is also not clear if the core (or even the complete particle) is composed of two (or more) particles and how they are connected. While from SEM the core/shell nature of the particles cannot be observed (Fig. 1B), this becomes clear in TEM (Fig. 2). On average, each particle is covered by five MoS_2_ shell layers. However, it can be seen that the shell is not homogeneously thick for one core/shell particle and that the shell thickness differs for different particles. On one side of a particle, there may be more layers of MoS_2_ than on the other side. This is nicely observable in Fig. 2C. The CNT network remains unaffected by the sulfidation treatment as the visible CNTs appear unchanged compared to untreated ones.^37^ An amorphous MoO_*x*_ layer remaining from the ALD deposition cannot be observed after the treatment, confirming a complete conversion to core/shell nanoparticles.^7^ Although the TEM sample preparation involved several minutes of ultrasonication to loosen the network, the particles are still in contact with the CNTs, as visible from the TEM images.

From HR TEM images (Fig. 2C) the lattice distances can be analyzed more locally, giving distances of 3.43 Å, 2.76 Å, 2.43 Å, 1.74 Å, and 1.40 Å for the core material. These values correspond to the (111̄), (102̄), (111), (113̄), and (131) planes of monoclinic MoO_2_.^33^ Monoclinic MoO_2_ was also observed after annealing of ALD-deposited MoO_*x*_ on CNTs in an argon atmosphere^7^ and also by sulfurization of MoO_3_ nanoflakes in a sulfidic argon atmosphere.^38^ The shell layer spacing of 6.5 ± 0.3 Å fits the (002) plane of hexagonal or the (003) plane of orthorhombic MoS_2_.^34,35^ As mentioned above, their *d*-values are very similar and it is therefore not possible to assign one modification with certainty. The deviation from the literature value of 6.14 Å for the bulk MoS_2_ can be caused by the curvature of the particle surface and resulting capillary effects in the (near-)surface region.^39^ Larger distances for MoS_2_ layered phases were also observed in literature.^22,38,40–42^ For example, Dahl-Petersen *et al.* described MoO_2_/MoS_2_ core/shell nanoparticles with larger MoS_2_ lattice spacing closer to the MoO_2_ surface of up to ≈7.3 Å as energetically possible due to the only weak van der Waals forces between the MoS_2_ sheets.^42^ Farther away from the MoO_2_ particle the MoS_2_ lattice spacing decreased.^42^ Additionally, another potential explanation could be the formation of the Chevrel phase Mo_6_S_8_ with a lattice spacing of 6.42 Å for the (101) plane (ICSD 252376).^43^ However, as XRD measurements (Fig. 1) of the here-presented triple-hybrid material only showed the MoS_2_ phase, this can be considered unlikely. The multiwall CNTs have a diameter of around 11 nm. An HR TEM image of such a multiwall CNT decorated with a core/shell particle is given in Fig. 2D. The spacing of the shell material is larger than that of the CNT. Measurements of the lattice spacing of the CNTs gave a value of ≈3.6 ± 0.2 Å which is very close to the (002) plane for graphitic carbon which has a spacing of 3.3 Å.^27^

With electron diffraction (diffractogram shown in Fig. 2A) the results of XRD and HR TEM can be supported. Two of the observed reflections, one at 6.5 Å and one at 4.7 Å, can only be explained by either MoS_2_ (ref. 34 and 35) or MoO_2_,^33^ respectively. All other observed reflections can be explained by both of these phases as well as graphitic carbon.^27^ The in the diffraction pattern visible amorphous rings originate from the carbon support. A differentiation between the orthorhombic and hexagonal MoS_2_ modification is not possible because, as mentioned above, their values are too similar.

**Fig. 3:**
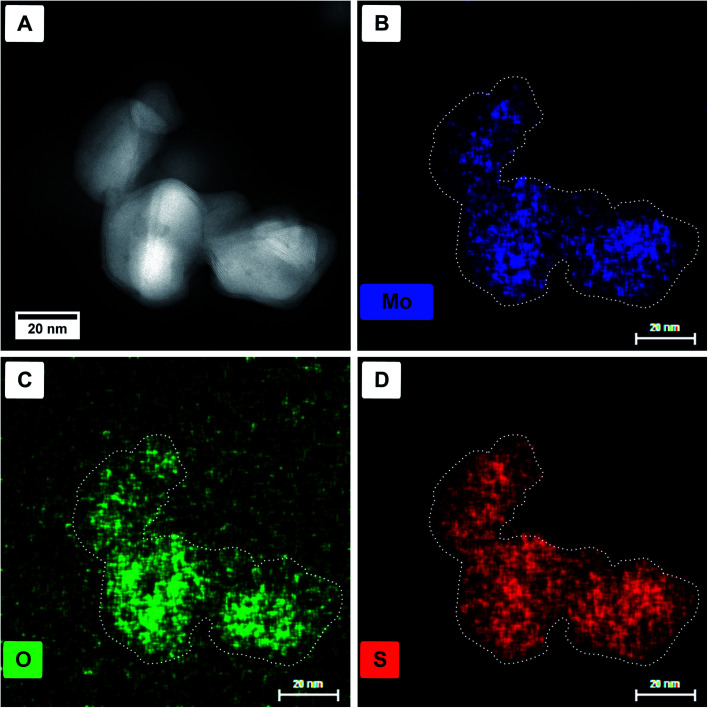
HAADF STEM images (A) and single-elemental EDX maps of molybdenum (B), oxygen (C), and sulfur (D) of the respective region. For better visibility, the outline of the particles is shown in the elemental maps as a dotted line.

Energy dispersive X-ray (EDX) spectroscopy was used to identify if the core material is a pure oxide and the shell solely sulfide. In Fig. 3, a HAADF STEM image (Fig. 3A) of a particle agglomerate, as well as the elemental EDX maps for molybdenum (Mo-K_α_, 17.48 keV, Fig. 3B), oxygen (O-K_α_, 0.53 keV, Fig. 3C), and sulfur (S-K_α_, 2.31 keV, Fig. 3D) are shown. For better visibility, the dotted outline of the particles was taken from panel A and laid over the elemental maps. The X-ray line energies are taken from literature.^44^ As can be seen, oxygen is only present in the core; however, molybdenum and sulfur seem to be distributed homogeneously. The main problem, in this case, is the presence of the Mo-L_α_ line appearing at 2.29 keV (ref. 44) and therefore overlapping with S-K_α_. Although for the mapping of the molybdenum content the Mo-K_α_ line is used, the overlap of Mo-L_α_ with S-K_α_ complicates the assignment of the elements.

**Fig. 4:**
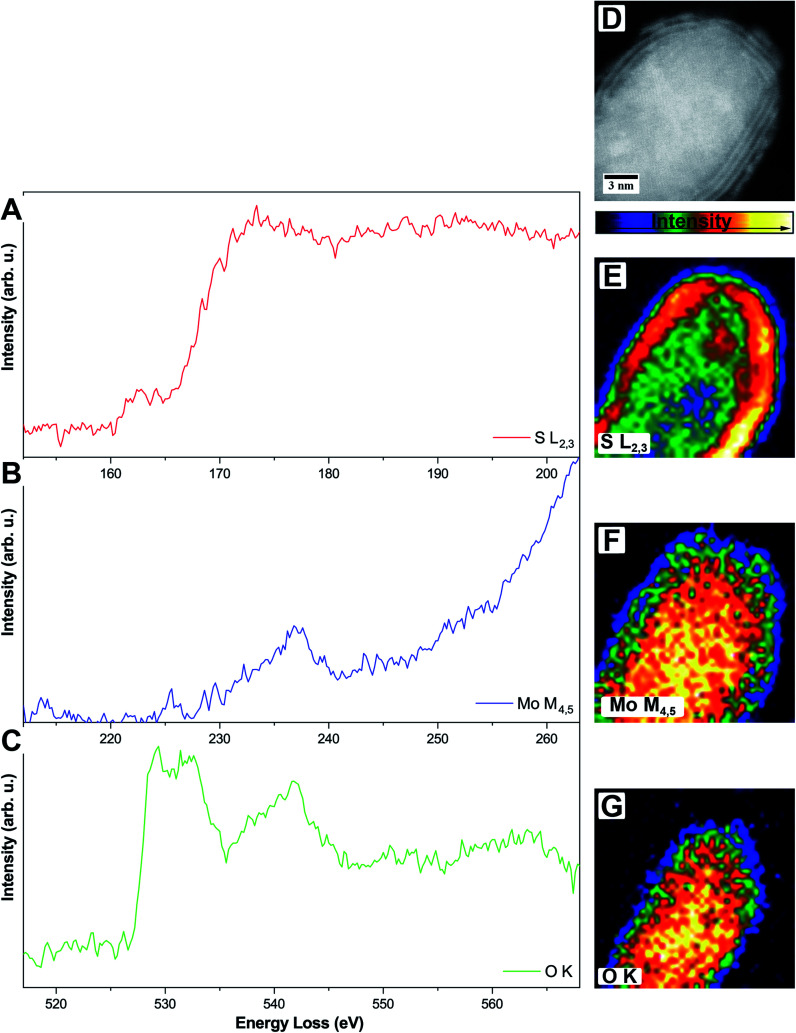
(A–C) Single-elemental EEL spectra of one MoO_2_/MoS_2_ core/shell particle, S-L_2,3_ (A), Mo-M_4,5_ (B), and O-K (C), respectively. (D–G) HAADF STEM image (D) and intensity-colored single-elemental EELS maps for S-L_2,3_ (E), Mo-M_4,5_ (F), and O-K (G), respectively.

To circumvent this problem, it is also possible to do elemental mapping with electron energy-loss spectroscopy (EELS). Here, molybdenum (Mo-M_4,5_, 227 eV) and sulfur (S-L_2,3_, 165 eV) were measured within one spectrum while oxygen (O-K, 532 eV) was measured separately due to the chosen dispersion of 0.1 eV per channel where a total energy loss region of about 200 eV can be covered. Single elemental EEL spectra are displayed in Fig. 4.

The S-L_2,3_ edge (Fig. 4A, red) shows the typical fine structure with an onset at around 165 eV, followed by a stronger rise of the intensity at ≈170 eV. These two are followed by a broad peak on which the Mo-M_4,5_ edge (Fig. 4B, blue) is superimposed. Due to the S-L_2,3_ edge, the intensity of the Mo signal is low but still shows a clear onset of the edge at 230 eV. It is a delayed edge with the peak maximum occurring above 250 eV. The O-K edge (Fig. 4C, green) of the core shows three dominant features in the electron loss region between 525 eV and 560 eV. The first two peaks at 529 eV and 532 eV are related to transitions to empty O-2p orbitals, which are hybridized with Mo-4d ones, while the third feature at 542 eV relates to the hybridization of O-2p with Mo-5sp orbitals. Fingerprint analysis using literature data, where the first two peaks were having a similar intensity followed by a third peak with lower intensity, indicates the formation of MoO_2_ instead of MoO_3_ (ICSD 166363)^45^ in accordance with the results from HR TEM.^46^

EELS elemental mappings of the three separate edges (Fig. 4D–G) show clear evidence, especially from the S-L_2,3_ mapping (Fig. 4E), that the shell of these particles gives the highest sulfur signal, while the oxygen signal (Fig. 4G) is limited only to the core. This further confirms that the shell of the core/shell particles consists of MoS_2_, while the core consists of molybdenum oxide.

When comparing different core/shell particles in the sample some conclusions can be drawn. First, all particles show distinct sulfide shells, which could be proven not only from HR TEM but also with the help of elemental analysis using EELS. Second, the shells around the particles are not homogeneously thick as described above. The thickness variations of the shells can be explained by the sulfidation synthesis itself, as the H_2_S gas stream is first reaching the samples from one side, facilitating the sulfidation starting from the side of the particles which do not adhere to the CNTs. Additionally, on certain facets of the oxide core growth of the sulfide can be preferential as observed by *in situ* measurements performed by Dahl-Petersen *et al.*^42^ Third, TEM, in general, only delivers 2D projection images (*i.e.*, not providing 3D information), which means that the real morphology of the core/shell particles cannot be determined in detail. For example, some of the particles look as if their core is composed of one or more smaller particles, where it is not clear if all the particles are covered by one sulfide shell or just lie on top of each other, and in other cases, the particles do not seem to be roundish but seem to have more complicated shapes (Fig. 2C).

Therefore, electron tomography was chosen to analyze the core/shell particles in more detail as it can give insights into the 3D morphology of the core/shell particles. As imaging mode, high angle annular dark-field (HAADF) STEM was used as the characteristic *Z*-contrast allows distinguishing between the two different materials, molybdenum oxide and molybdenum sulfide, solely by their intensity in the images. It has to be mentioned that in the presented case, the observed intensity difference of core and shell is mainly caused by less material and, therefore, less scattering of the shell, resulting in a lower intensity compared to the core material. However, working in HAADF has the additional advantage of minimizing diffraction contrast, which would lead to difficulties in the interpretation and processing of the tomography series. Also, it should be mentioned that particles and areas which could be rotated around their long axis were chosen for tomography to avoid signal interference. To further investigate the influence of the carbon coating of the TEM grid onto the 3D reconstruction, samples on continuous and lacey carbon, respectively, were analyzed.

**Fig. 5:**
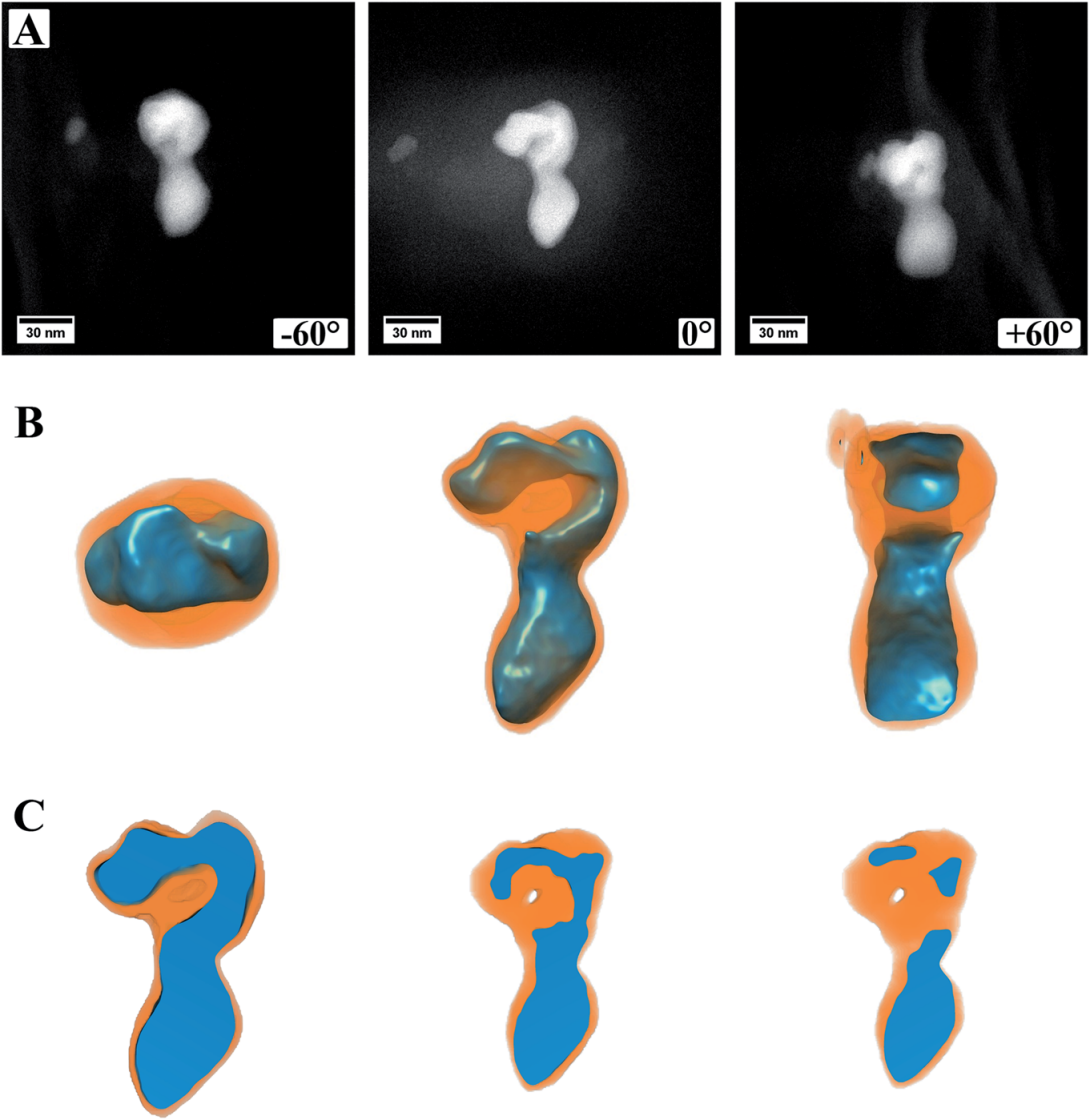
Images and reconstructed volume of a MoO_2_/MoS_2_ core/shell particle from the TEM sample prepared on a grid coated with continuous carbon. (A) HAADF STEM images from the tilt series of one area used for reconstruction. Three different angles are shown. (B) The reconstructed volume of the core/shell particle displayed in different orientations. (C) Cuts through the reconstructed volume showing a solid oxide core but a small hole in the sulfide shell.

Fig. 5 (ESI Video S1†) shows a summary of the electron tomography of one particle of the sample, where a continuous carbon coating on the TEM grid was used. Panel A shows three exemplary HAADF STEM images, which were obtained during the acquisition of the tilt series, more precise at the maximum angles ±60° as well as 0°. From the images alone, it is not clear if the particle is one single particle with an irregular shape or if two (or more) particles are lying on top of each other. It is also not discernible if, for example, the cores of these particles are connected or not. Panel B shows the successful 3D reconstruction of the particle. For this, the different intensities in the HAADF images were modeled and computed in an iterative approach, fitting two different material factors to the original data. After 3D reconstruction it is possible to tilt the particle in different directions, judging its shape from different directions (Fig. 5B). This allows investigating whether the particle is one single particle or composed of two or more particles. The shown feature can now be easily identified as a single particle with a continuous core, which has an elongated and irregular shape. Most likely, this particle evolved by coalescence of two or more particles, leading to its shape.

It is also possible to cut into the reconstructed volume (Fig. 5C) to analyze the inside of the particle. In the presented case, it can be proven that the oxide core is made of dense material without pores, and only a small hole appears within the shell. From the cut at roughly the center of the particle (Fig. 5C, left image) it also becomes evident that the sulfide shell is not homogeneously thick around the whole core, as already observed from (S)TEM images. This can be, as mentioned already, traced back to the synthesis conditions with the reactive gas flow starting the reaction from one side and the particles being attached to the carbon nanotubes on the other side.

In Fig. 5A, the image from a 0° tilting angle (middle) shows a brighter contrast around the particle, which is missing in the other images. This is related to carbon contamination/re-deposition by the high-intensity electron beam and gets stronger the longer or more often a certain area is illuminated. For 0°, this is more pronounced as it is the angle at which more often images were taken. To minimize carbon contamination, the samples were plasma cleaned before inserting into the TEM, which strongly reduced the effect of carbon contamination. However, as can be seen, it cannot be avoided entirely. For high tilting angles of ±60°, focusing the images becomes increasingly difficult, which is related to a too low depth of focus at the used camera length and could be increased by lowering the convergence angle. However, the successful reconstruction indicates that both effects are not affecting the outcome of the 3D reconstruction strongly. The success of the reconstruction can easily be judged by comparing modeled micrographs based on the 3D model to the original data and computing their difference.

**Fig. 6:**
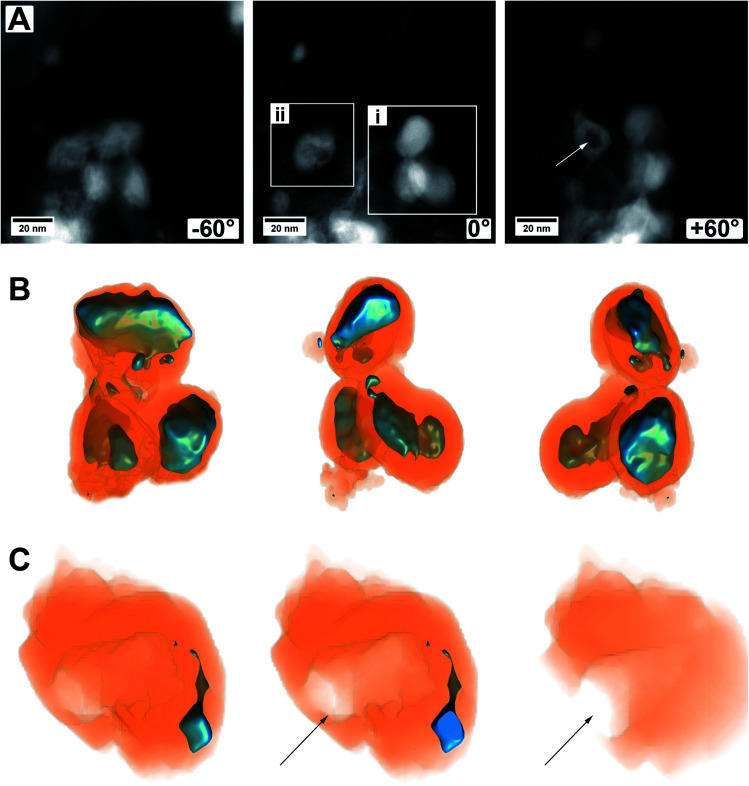
Images and reconstructed volume of a MoO_2_/MoS_2_ core/shell particle from the TEM sample prepared on a grid coated with lacey carbon. (A) HAADF STEM images from the tilt series of one area used for reconstruction. Three different angles are shown. (B) The reconstructed volume of area (i) of several core/shell particles displayed in different orientations. (C) Cuts through the reconstructed volume of area (ii) showing a mainly empty sulfide shell with an opening to the outside, marked by an arrow.

Another set of electron tomography on some MoO_2_/MoS_2_ core/shell particles is shown in Fig. 6 (ESI Video S2†), where the sample was prepared on a TEM grid coated with lacey carbon. HAADF STEM images of an area with several core/shell particles at different tilt angles are shown in Fig. 6A. On the right in the image with tilting angle 0°, a group of particles is displayed (area i, ESI Video S2†, area i), which seem to lie on each other but not being connected. On the left, a particle with a different contrast in the middle is displayed (area ii, ESI Video S2†, area ii), but the HAADF image alone is not explaining it (*e.g.*, if the particle is hollow, or if it is not a real core/shell particle, *etc.*).

A reconstruction of area (i), displayed at different viewing directions, is given in Fig. 6B, proving that there are three distinct core/shell particles in close vicinity to each other. The view at different tilt angles shows that the shape of the core is not always smooth and roundish but partly possessing protrusions or smaller oxide particles which are not in contact with the primary core particle. This could be due to the relatively short annealing time of 15 min and could be removed most likely by prolonged annealing treatment times, that is, enhancing particle growth by Ostwald ripening. Possibly, the developing sulfur shell is preventing the particles from further coalescence.

Fig. 6C shows several cuts into the particle of area (ii). The reconstruction now clearly demonstrates that only a small part of this area is made from molybdenum oxide. From the cuts, it becomes obvious that the main part of this particle is hollow and even shows an opening to the outside (marked by an arrow). This indicates that a part of the oxide core was removed either during the synthesis of the material (full sulfurization) or later during ultrasonication for (S)TEM sample preparation. For example, there could have been a weak or thin point in the shell, or the shell could have been even missing completely, possible at areas of the particles which were (and still are) in contact with the carbon nanotube network. Whatever the case may be, this observation gives a hint of why the cycling stability of the as-prepared material may not be as good and can give a hint for one degradation pathway of the material.

In conclusion, our work showed the successful investigation of metal oxide/metal sulfide core/shell particles within carbon nanotubes (triple hybrid) in up to three dimensions. Electron microscopic investigations with EDX and EELS, as well as high-resolution TEM, gave insights into the chemical and structural composition of the hybrid materials. By using HAADF imaging in STEM and tomographic reconstruction of the tilt series, the separation of the oxide core and sulfide shell could be done by solely using the contrast in the images, and the results give insights into the 3D morphology of the particles. Doing this, first indications of possible degradation pathways of the material can be given. The use of TEM grids with different supports shows no influence on the quality of the reconstructions.

## 4 Conclusions

This work shows the successful preparation of MoO_2_/MoS_2_–CNT core/shell hybrid materials, which can be used as electrodes in, for example, Li-ion batteries or supercapacitors. Due to the complexity of the materials, a thorough characterization of it is needed, which was performed by combining different (S)TEM techniques. Elemental analysis of the material was successfully performed by EELS measurements. 3D electron tomography has been effectively used to investigate the MoO_2_/MoS_2_ core/shell particles by reconstruction of the two distinct materials. First insights into the set-up of the particles, that is, the thickness of the shell, contact between core and shell, as well as about possible degradation mechanisms could be revealed. The study presents a powerful imaging technique that can be employed in future studies to correlate hybrid material nanostructure with electrochemical energy storage performance. It is proposed to use identical location measurements to compare the nanostructure of pristine samples with that of cycled samples to understand degradation mechanisms more clearly.

## Conflicts of interest

There are no conflicts to declare.

## Supplementary Material

NA-003-D0NA00806K-s001

NA-003-D0NA00806K-s002

NA-003-D0NA00806K-s003

NA-003-D0NA00806K-s004

## References

[cit1] Fleischmann S., Tolosa A., Presser V. (2018). Chem.–Eur. J..

[cit2] Seok D., Jeong Y., Han K., Yoon D. Y., Sohn H. (2019). Sustainability.

[cit3] Sun H., Zhu J., Baumann D., Peng L., Xu Y., Shakir I., Huang Y., Duan X. (2019). Nat. Rev. Mater..

[cit4] Fleischmann S., Mitchell J. B., Wang R., Zhan C., Jiang D.-e., Presser V., Augustyn V. (2020). Chem. Rev..

[cit5] Qiu S., Gu H., Lu G., Liu J., Li X., Fu Y., Yan X., Hu C., Guo Z. (2015). RSC Adv..

[cit6] Nam I., Kim N. D., Kim G.-P., Park J., Yi J. (2013). J. Power Sources.

[cit7] Fleischmann S., Zeiger M., Quade A., Kruth A., Presser V. (2018). ACS Appl. Mater. Interfaces.

[cit8] Noerochim L., Wang J.-Z., Chou S.-L., Li H.-J., Liu H.-K. (2010). Electrochim. Acta.

[cit9] Um J. H., Lim J., Hengge K., Scheu C., Yoon W.-S., Lee J.-K., Sung Y.-E. (2019). Composites, Part B.

[cit10] Stephenson T., Li Z., Olsen B., Mitlin D. (2014). Energy Environ. Sci..

[cit11] Cook J. B., Kim H.-S., Lin T. C., Lai C.-H., Dunn B., Tolbert S. H. (2017). Adv. Energy Mater..

[cit12] Budak Ö., Srimuk P., Tolosa A., Fleischmann S., Lee J., Hieke S. W., Frank A., Scheu C., Presser V. (2019). Batteries Supercaps.

[cit13] Brown E., Park S.-H., Elangovan A., Yuan Y., Kim J., Sun X. S., Zhang X., Wang G., Li J. (2018). Electrochim. Acta.

[cit14] Li G., Wang X., Ma X. (2013). J. Energy Chem..

[cit15] Zhou X., Wu G., Wu J., Yang H., Wang J., Gao G., Cai R., Yan Q. (2013). J. Mater. Chem. A.

[cit16] Zeiger M., Ariyanto T., Kruner B., Peter N. J., Fleischmann S., Etzold B. J. M., Presser V. (2016). J. Mater. Chem. A.

[cit17] Boukhalfa S., Evanoff K., Yushin G. (2012). Energy Environ. Sci..

[cit18] Batenburg K. J., Bals S., Sijbers J., Kübel C., Midgley P. A., Hernandez J. C., Kaiser U., Encina E. R., Coronado E. A., Van Tendeloo G. (2009). Ultramicroscopy.

[cit19] Leary R. K. and Midgley P. A., in Springer Handbook of Microscopy, ed. P. W. Hawkes and J. C. H. Spence, Springer International Publishing, Cham, 2019, p. 2

[cit20] Weyland M., Midgley P. A. (2004). Mater. Today.

[cit21] Midgley P. A., Ward E. P. W., Hungría A. B., Thomas J. M. (2007). Chem. Soc. Rev..

[cit22] Zhang Y., Tao H., Ma H., Du S., Li T., Zhang Y., Li J., Yang X. (2018). Electrochim. Acta.

[cit23] Chen Z., Cummins D., Reinecke B. N., Clark E., Sunkara M. K., Jaramillo T. F. (2011). Nano Lett..

[cit24] Jin B., Zhou X., Huang L., Licklederer M., Yang M., Schmuki P. (2016). Angew. Chem., Int. Ed..

[cit25] Xu Z., Wang T., Kong L., Yao K., Fu H., Li K., Cao L., Huang J., Zhang Q. (2017). Part. Part. Syst. Charact..

[cit26] Zhang Z., Zhao H., Teng Y., Chang X., Xia Q., Li Z., Fang J., Du Z., Świerczek K. (2018). Adv. Energy Mater..

[cit27] Wyckoff R. W. G. , Crystal Structures, Interscience Publishers, New York, New York, Second edn, 1963, vol. 1

[cit28] MessaoudiI C., Boudier T., Sorzano C. O. S., Marco S. (2007). BMC Bioinf..

[cit29] Gilbert P. (1972). J. Theor. Biol..

[cit30] Lakshminarayanan A. V., Lent A. (1979). J. Theor. Biol..

[cit31] Gregor J., Benson T. (2008). IEEE Transactions on Medical Imaging.

[cit32] Zürner A., Döblinger M., Cauda V., Wei R., Bein T. (2012). Ultramicroscopy.

[cit33] Bolzan A., Kennedy B., Howard C. (1995). Aust. J. Chem..

[cit34] Bronsema K. D., De Boer J. L., Jellinek F. (1986). Z. Anorg. Allg. Chem..

[cit35] Takéuchi Y., Nowacki W. (1964). Schweiz. Mineral. Petrogr. Mitt..

[cit36] Scherrer P. (1918). Nachr. Ges. Wiss. Goettingen, Math.-Phys. Kl..

[cit37] Branca C., Frusteri F., Magazù V., Mangione A. (2004). J. Phys. Chem. B.

[cit38] Kumar P., Singh M., Sharma R. K., Reddy G. B. (2016). Mater. Res. Express.

[cit39] Maier J. (2013). Angew. Chem., Int. Ed..

[cit40] Dong Y., Jiang H., Deng Z., Hu Y., Li C. (2018). Chem. Eng. J..

[cit41] Zhang Q., Bai H., Zhang Q., Ma Q., Li Y., Wan C., Xi G. (2016). Nano Res..

[cit42] Dahl-Petersen C., Šarić M., Brorson M., Moses P. G., Rossmeisl J., Lauritsen J. V., Helveg S. (2018). ACS Nano.

[cit43] Chae M. S., Heo J. W., Lim S.-C., Hong S.-T. (2016). Inorg. Chem..

[cit44] Bearden J. A. (1967). Rev. Mod. Phys..

[cit45] Sitepu H. (2012). Powder Diffr..

[cit46] Wang D., Su D. S., Schlögl R. (2004). Z. Anorg. Allg. Chem..

